# Pro-Apoptotic Activity of New Honokiol/Triphenylmethane Analogues in B-Cell Lymphoid Malignancies

**DOI:** 10.3390/molecules21080995

**Published:** 2016-07-30

**Authors:** Aleksandra Mędra, Magdalena Witkowska, Agata Majchrzak, Barbara Cebula-Obrzut, Michael Y. Bonner, Tadeusz Robak, Jack L. Arbiser, Piotr Smolewski

**Affiliations:** 1 Department of Experimental Hematology of Medical University of Lodz, Ciołkowskiego 2 Street, Lodz 93-510, Poland; olajeske@o2.pl (A.M.); magdamalicka@gmail.com (M.W.); majchrzak_agata@o2.pl (A.M.); barbara_cebula@wp.pl (B.C.-O.); 2 Department of Hematology of Medical University of Lodz, Ciołkowskiego 2 Street, Lodz 93-510, Poland; robaktad@csk.umed.lodz.pl; 3 Winship Cancer Institute, Emory University School of Medicine, 101 Woodruff Cir, Atlanta, GA 30322, USA; mybonne@emory.edu (M.Y.B.); jarbise@emory.edu (J.L.A.); 4 Atlanta Veterans Administration Hospital, 1670 Clairmont Road, Decatur, GA 30033, USA

**Keywords:** honokiol analogues, CLL, ALL, BL, DLBCL, MM

## Abstract

Honokiol and triphenylmethanes are small molecules with anti-tumor properties. Recently, we synthesized new honokiol analogues (HAs) that possess common features of both groups. We assessed the anti-tumor effectiveness of HAs in B-cell leukemia/lymphoma cells, namely in chronic lymphocytic leukemia (CLL) cells ex vivo and in pre-B-cell acute lymphoblastic leukemia (Nalm-6), Burkitt lymphoma (BL; Raji), diffuse large B-cell lymphoma (DLBCL; Toledo) and multiple myeloma (MM; RPMI 8226) cell lines. Four of these compounds appeared to be significantly active against the majority of cells examined, with no significant impact on healthy lymphocytes. These active HAs induced caspase-dependent apoptosis, causing significant deregulation of several apoptosis-regulating proteins. Overall, these compounds downregulated Bcl-2 and XIAP and upregulated Bax, Bak and survivin proteins. In conclusion, some of the HAs are potent tumor-selective inducers of apoptosis in ex vivo CLL and in BL, DLBCL and MM cells in vitro. Further preclinical studies of these agents are recommended.

## 1. Introduction

Honokiol (HNK) is an active small biphenolic compound purified from *Magnolia* spp. Dried magnolia stem bark is well known and widely used in traditional Chinese and Japanese medicine in the treatment of many aliments (nervous disorders, anxiety, fever, thrombotic stroke and gastrointestinal symptoms) [1,2]. This plant-derived compound due to its pharmacological properties (antibacterial, antifungal, antioxidant, anti-inflammatory, anti-thrombotic, anti-allergic and anxiolytic) has attracted a great deal of research interest [3,4,5,6,7,8]. Recent studies show that HNK can play an important role as an anti-tumor agent, acting as an inhibitor of cell proliferation and growth and leading to cell apoptosis. Moreover, HNK counters metastasis and suppresses angiogenesis [9,10].

HNK has attracted attention as a potential antineoplastic agent because it has demonstrated broad activity against multiple types of tumors [11,12,13]. Studies assessing HNK’s mechanisms of action concluded that HNK induced apoptosis via cytochrome c release and effector caspase activation [11,12,14]. The precise mechanism remains still not fully discovered, but according to recent knowledge, it seems to be associated with changes in the expression of Bcl-2 and Mcl-1 proteins [15,16]. Moreover, exposure to HNK leads to inhibition of NF-κB, as a result of the reduction of the nuclear NF-κB level with the concurrent increase in cytoplasmatic level [17,18]. In addition, pretreatment of cells in the presence of HNK leads to inhibition of Akt/phosphoinositide 3-kinase (PI3K) and mitogen-activated protein kinase (MAPK) signaling [11]. In vitro experiments showed that HNK acts against skin, colon, lung, pancreatic and breast cancer cells and against cell lines, e.g., derived from human lymphoid leukemia (Molt4) cells, human colorectal carcinoma (RKO), human squamous lung cancer (CH27) or human promyelocytic leukemia (HL-60) [10,11,12,17,19,20,21,22,23]. It has a stronger effect on chronic lymphocytic leukemia (CLL) and multiple myeloma (MM) cells, rather than on normal mononuclear lymphocytes [10,12,13]. In vivo studies confirmed the proapoptotic and antineoplastic activity of HNK on SVR angiosarcoma, breast cancer in nude mice and in a human A549 lung cancer xenograft model [11,24,25].

One of the barriers to the development of HNK as a therapeutic is that it is difficult to synthesize in large quantities. We have recently demonstrated that another class of small molecules, triphenylmethanes, have activity against tumor cells, in part through NADPH oxidase inhibition. In order to overcome the synthetic obstacles and potentially introduce novel modes of activity, we synthesized novel honokiol analogues (HAs) that contain features of both honokiol and triphenylmethanes. We tested these analogues against freshly-isolated cells from CLL patients, as well as a panel of cell lines from common B-cell malignancies. Of the seven analogues we synthesized, four were broadly active against both patient isolates and cell lines. These compounds deserve further preclinical evaluation as novel therapies for B-cell malignancies, many of which are currently incurable.

## 2. Results

**Figure 1:**
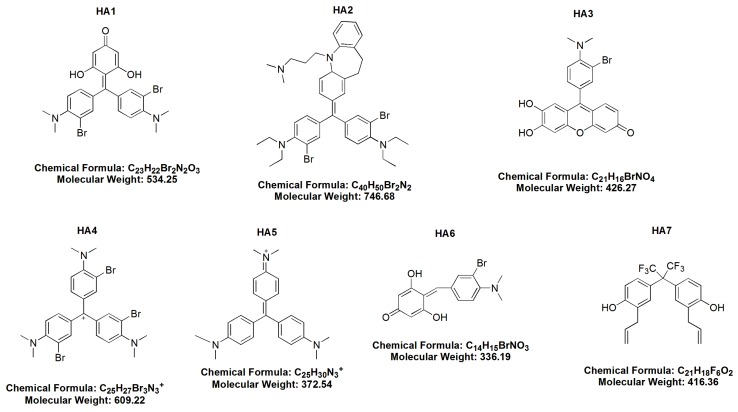
The chemical structures of all examined honokiol analogues (HA1–HA7).

The chemical structures of the examined HAs are shown in Figure 1.

### 2.1. Cytotoxicity of HAs

HAs were tested in concentrations 0.1–10 μM, then the minimal doses that triggered a significant increase in cytotoxicity and apoptosis at the 48 h time point were chosen for further experiments. The level of cytotoxicity assessed by PI staining strongly correlated with AI evaluated by the Ann-V assay (R = 0.86, *p* < 0.001); therefore, further experiments were based on CAI values.

HA 1 triggered significant apoptosis starting from the dose of 5 μM, with minimal significant CAI (msCAI) 12.5%; *p* = 0.043 (IC_50_ 10 μM). In Raji cells, msCAI was 17% at a dose of 0.5 μM; *p* = 0.025 (IC_50_ 2.5 μM). In Toledo cells, msCAI was 28% (at 0.1 μM); *p* = 0.007 (IC_50_ 0.5 μM) and RPMI 8226 (msCAI 38.1% at 0.5 μM; *p* < 0.001, IC_50_ 0.5 μM) cells (Table 1).

HA 2 in CLL cells induced both msCAI 21.9% at 2.5 μM; *p* < 0.001 (IC_50_ 5 μM). In Raji cells, msCAI was 22.1% (at 1 μM); *p* = 0.025, with IC_50_ 2.5 μM. The highest anti-tumor effect of HA 2 was found for Toledo (msCAI 32% at 0.25 μM; *p* < 0.001, IC_50_ 0.5 μM) and RPMI 8226 (msCAI 25.7% at 0.1 μM; *p* = 0.007, IC_50_ 0.5 μM) cell lines (Table 1).

HA 4 in CLL cells induced msCAI 15.8% (2.5 μM); *p* = 0.027 (IC_50_ 10 μM). In Raji cells, msCAI was 27.4% (at 2.5 μM); *p* = 0.015 (IC_50_ 7.5 μM). Toledo and RPMI 8226 showed msCAI at the same dose of 1 M; msCAIs were 35.2% and 18.4%, respectively; *p* < 0.001 (IC_50_ 2.5 μM and 7.5 μM, respectively) (Table 1).

HA 5 in CLL cells triggered msCAI 23% (*p* = 0.012) at 2.5 μM (IC_50_ 5 μM). In Raji model, msCAI was 34.7 % at 0.1 μM; *p* < 0.001 (IC_50_ 0.25 μM). In Toledo, msCAI was 30.3% (at 0.25 μM); *p* < 0.001 (IC_50_ 0.5 μM). In RPMI 8226 cells, msCAI was 26.5% (at 0.5 μM); *p* < 0.001 (IC_50_ 1 μM) (Table 1).

After a series of preliminary experiments, three HAs (HA 3, 6 and 7) were rejected because they did not significantly affect the examined malignant cells. 

### 2.2. Mechanisms of HA Action

**Figure 2:**
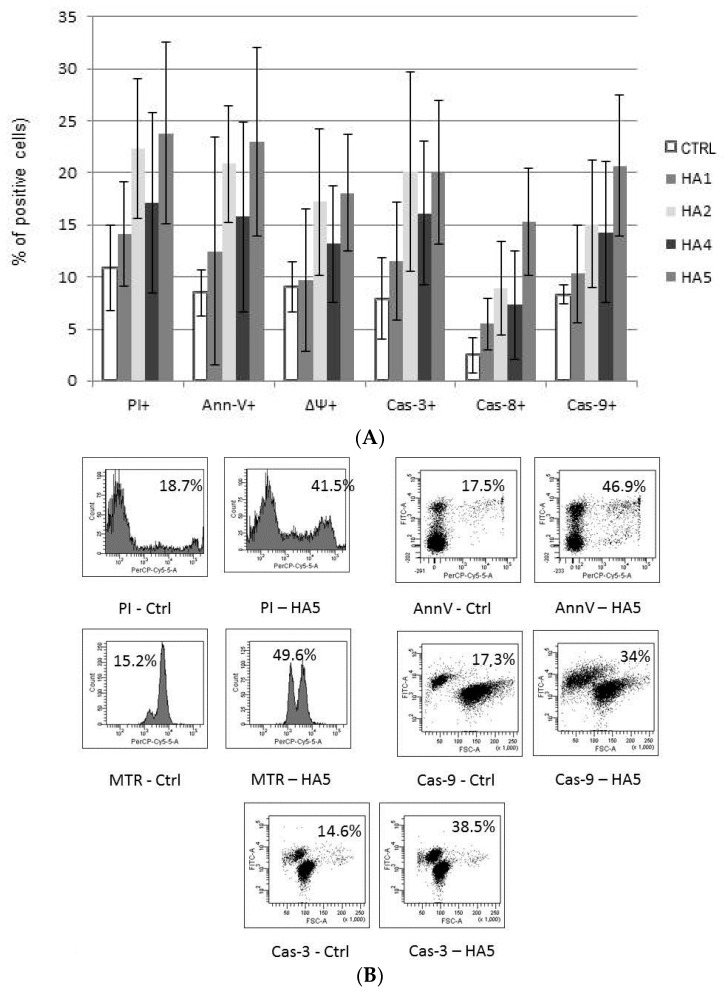
(**A**) The mechanisms of action of honokiol and honokiol analogues (HAs) 1, 2, 4 and 5. All of those HAs significantly enhanced the activation of caspases-3 (Cas-3) and -9 (Cas-9), as well as induced the drop of the mitochondrial potential (MTR, ΔΨ) in chronic lymphocytic leukemia (CLL) cells after 48 h of incubation. Overall cytotoxicity, as measured by the propidium iodide (PI) assay, is also shown. The data compensated by appropriate untreated controls are presented; (**B**) Representative flow cytometry plots for propidium iodide (PI) and apoptotic parameters: chronic lymphocytic leukemia (CLL) cells, 48 h of incubation with honokiol analogue 5 (HA 5) vs. untreated controls.

**Figure 3:**
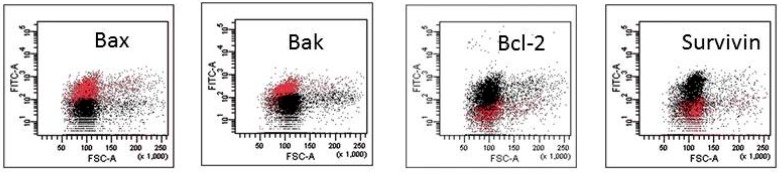
Representative flow cytometry plots for apoptosis-regulating proteins, the expression of which has been significantly changed after 48 h of incubation of Raji cells with honokiol analogue 5 (HA 5) vs. untreated controls.

The mechanism of action of HAs 1, 2, 4 and 5 was caspase-dependent apoptosis, triggered through both the TNF receptor and mitochondrial pathways. All of those HAs significantly enhanced the activation of caspases-3, -8 and -9, as well as induced the drop of mitochondrial potential in CLL cells. Similarly, in the cell lines tested, the cytotoxic effect was mainly dependent on caspase-dependent apoptosis (Figure 2A). The representative flow cytometry plots for PI and particular apoptotic parameters in CLL cell cultures after 48 h of incubation with HA 5 vs. untreated controls are demonstrated in Figure 2B. Similarly, the representative flow cytometry plots for apoptosis-regulating proteins, the expression of which has been significantly changed, are shown in Figure 3 (an example for after 48 h incubation of Raji cells with HA 5 vs. untreated controls).

The expression of several apoptosis-regulating proteins, including the Bcl-2 family, the IAP family and IAP antagonists, after treatment with HAs 1, 2, 4 and 5 was evaluated, and detailed results are shown in Table 2A–D. In general, in CLL cells, most of them significantly downregulated Bcl-2, Mcl-1, cIAP-1, XIAP and Smac/DIABLO proteins and upregulated survivin. In Raji cells, significant Bcl-2 downregulation, with upregulation of Bax and Bak proteins, was found. In Toledo cells, decreased expression Bcl-2 and XIAP and overexpression of Bax and survivin were observed. In RPMI 8226 cells, all of those HAs significantly downregulated Bcl-2 and XIAP and upregulated Bax, Bak and survivin proteins. In contrast, Nalm-60 cells were resistant to all of those HAs in regard to the change in the examined protein expression (Table 2A–D).

**Figure 4:**
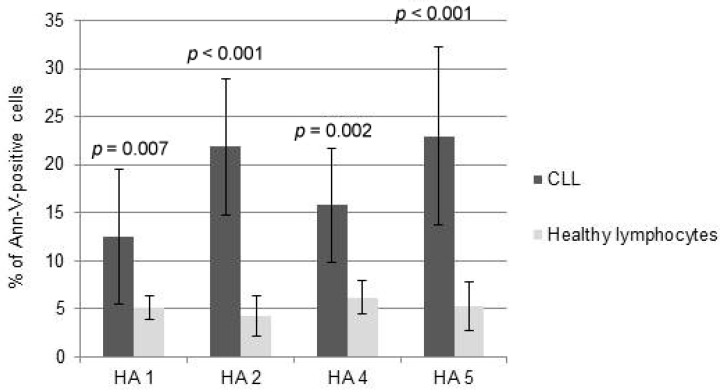
Comparison between the cytotoxicity of honokiol analogues (HAs) 1, 2, 4 and 5 measured as propidium iodide towards healthy lymphocytes and chronic lymphocytic leukemia (CLL) cells.

**Figure 5:**
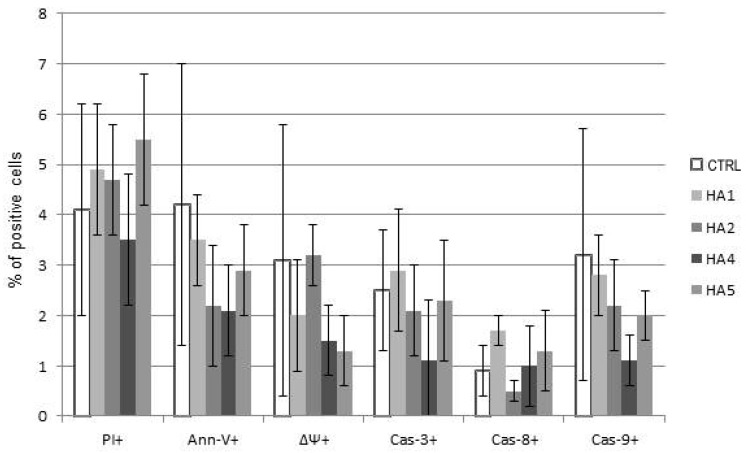
The mechanisms of action of honokiol analogues (HAs) 1, 2, 4 and 5 on healthy lymphocytes. Results of propidium iodide (PI) staining and assessed apoptotic parameters annexin-V (Ann-V), drop of mitochondrial potential (ΔΨ), caspases-3, -8 and -9 (Cas-3, -8 and -9) are shown. The data compensated by appropriate untreated controls are shown.

Interestingly, the comparison between the cytotoxicity of HAs towards normal PBMCs and CLL cells demonstrated that HAs did not trigger significant levels of apoptosis of healthy lymphocytes at doses inducing msCAI (Figure 4). Pro-apoptotic mechanisms were almost not triggered; HAs were used in doses inducing active cell death of tumor cells (Figure 5).

## 3. Discussion

In this study, we synthesized novel analogues that have honokiol-like and triphenylmethane-like properties in the same molecule. Our structure activity relationship begins with two disparate pharmacophores, the triphenylmethanes, as exemplified by gentian violet, and polyphenols, as exemplified by honokiol. These classes have differing mechanisms of action, and the triphenylmethanes appear to have mitochondrial localization due to the presence of charged alkylamino groups, while the polyphenols do not. This is an initial attempt to blend the two classes together in order to potentially obtain more versatile molecules. Halogenation was added in order to increase lipophilicity and slow metabolism. Of the seven analogues we studied, four of them had potent activity against multiple patient-derived CLL isolates and the majority of B-cell lymphoid cell lines. Our present study reveals for the first time the strong pro-apoptotic activity of four examined HAs (HA 1, HA 2, HA 4 and HA 5) in both CLL and B-cell lymphomas, with less evident impact for the survival of ALL cells (Nalm-6). All of those active compounds have a triphenylmethane structure, as opposed to HA 6 and HA 7, which are not triphenylmethanes. The inactive HA 3 is a triphenylmethane, but the structure is a planar ring, and it may be that fixing the ring is a tricyclic conformation blocking the activity of the compound. 

HNK has shown activity against CLL, potentiating apoptosis in the presence of the antiapoptotic cytokine IL-4. It was reported that CLL cells are more sensible to HNK than PBMCs. Exposure of CLL cells to HNK leads to them undergoing apoptosis upon caspase activation and modulating the expression of key apoptotic regulatory proteins Bcl-2 protein and IAP families [12]. In MM cells, HNK promoted apoptosis in both dexamethasone-sensitive and -resistant cell lines and promoted apoptosis in the presence of protective factors, including IL-8, IGF-1 and bone marrow stromal cells. Furthermore, preincubation of CLL and MM cells in the presence of the broad spectrum caspase inhibitor z-VAD-fmk almost completely inhibits the cleavage of pro-caspase 3 and, as a result, causes the inhibition of apoptosis. That confirms that HNK induces caspase-dependent apoptosis [12,13].

Several trends were observed in this study. The active compounds appeared to cause similar downregulation of Mcl-1, bcl2, XIAP, cIAP-1 and cIAP-2 in CLL cells. No activity was seen on Bax and Bak in CLL cells. On the other hand, active analogues increased Bax and Bak in most B-cell lymphoid cell lines. Notably, survivin was upregulated by active analogues in CLL cells, as well as B-cell lymphoid cell lines. The only resistant cell line was Nalm-6, a pre-B-cell ALL cell line. Of interest, Nalm-6 is deficient in the repair gene MSH2 and is highly proficient in homologous recombination [26]. Deficiency in MSH2 is found in a small fraction of ALL and is associated with resistance to thiopurines and increased sensitivity to the alkylating agent melphalan [27]. Our data suggest that cells deficient in MSH2 may be more resistant to our analogues than other B-cell malignancies. Moreover, the lack of efficacy in the Nalm-6 cells may be a useful biomarker. This may suggest the usefulness of immunohistochemical staining of tumor cells for mismatch repair proteins. Those tumors that are negative for mismatch repair are probably not appropriate candidates for therapy with HAs.

Finally, survivin upregulation appears to be a common response to treatment with our active analogues. A recent study demonstrated that high levels of reactive oxygen downregulate survivin expression in estrogen receptor (ER)-positive breast cancer cells. These ER-positive tumors demonstrated elevated levels of Nox-1 and p67phox, indicating the role of reactive oxygen in estrogen-induced tumors. Overexpression of survivin in this context led to decreased tumor volume in breast tumor xenografts. The induction of survivin in both CLL isolates and B-cell leukemia/lymphoma cells could be consistent with the findings of reactive oxygen downregulating survivin. Survivin upregulation could also serve as a biomarker for the efficacy of these novel analogues [28].

In part, the HNK-induced apoptosis is also caspase-independent. It was observed that HNK caused apoptosis even when the level of caspase-8 and -3 was low [12,13]. In caspase-independent activation, HNK leads to the release of apoptosis inducing factor (AIF) from mitochondria to the cytosol and nucleus with concomitant condensation of chromatin and cell death as a consequence in MM cells. According to our data, in regard to HAs, the mechanism of their anti-apoptotic anti-tumor activity appears to depend on both mitochondrial caspase-activation pathways, since caspases-9 and -3 are activated concurrently with a decline of mitochondrial potential [12,29]. However, we found also activation of the external pathway in response to HA 1, 2, 4 or 5 in most examined tumor cells.

The Bcl-2 family proteins play a critical role in apoptosis, acting as either promoters (Bax, Bak) or inhibitors (Bcl-2, Bcl-xL) of programmed cell death. A high ratio of Bcl-2/Bax and increased expression of Mcl-1 are found in hematologic malignances. Furthermore, high expression of Mcl-1 is associated with resistance to chemotherapy (chlorambucil and fludarabine (FA) in CLL) [12,30]. HNK-mediated apoptosis is activated by the increase in the levels of proapoptotic proteins (Bax, Bak) and the decrease in the levels of Bcl-2 and Mcl-1 (antiapoptotic proteins). The alternation in the expression of the Bcl-2 family mediated by HNK varies in different cancer cells [12,13]. Apoptosis in HNK-treated CLL cells is led by upregulation of Bax and downregulation of Mcl-1 [12,13,28]. Mcl-1 cleavage is characteristic for MM cells incubated with HNK, but the levels of Bak, Bax, Bcl-2, Bcl-xL and Bid were not altered [13]. In the CH27 human squamous lung cancer cell line, HNK induces apoptosis by the reduction of Bcl-xL protein expression [21,30]. In our study, we observed changes in the expression of several apoptosis-regulating proteins from the Bcl-2 (mainly Bax, Bcl-2 and XIAP) or IAP families (mainly XIAP and survivin) in different types of tumor cells treated with HA 1, 2, 4 and 5. 

Interestingly, lowest concentrations of those HAs induce apoptosis of tumor cells, but did not significantly affect the viability of normal lymphocytes. 

In conclusion, these data indicate that some HAs are potent tumor-selective inducers of apoptosis in ex vivo CLL cells and in the in vitro model of BL, DLBCL and MM. Those HAs should be examined for further clinical application, either as single agents or in combination with other anti-cancer drugs. 

## 4. Material and Methods

We performed ex vivo experiments on CLL cells (obtained from 31 previously untreated CLL patients). The CLL group consisted of 11 women and 20 men, median age 71.1 (range 50–84 years). There were 15 patients in stage 0–I according to Rai’ classification, 8 patients in stage II and 8 in stage III–IV. Eighteen patients were in stable and 13 in progressive disease. Two out of 31 patients had 17p13 deletion, 3/31 had 11q region deletion or translocation. In CLL cells, 8/31 were ZAP-70-positive, and 9/31 showed CD38-positivity.

Moreover, in vitro studies on cell lines were derived from pre-B-cell acute lymphoblastic leukemia (Nalm-6), Burkitt lymphoma (BL) (Raji), diffuse large B-cell lymphoma (DLBCL) (Toledo) and MM (RPMI 8226) cells (all cell lines from American Type Culture Collection ATCC, Manassas, VA, USA). Additionally, cells obtained from 15 healthy volunteers were also treated. The study was performed in accordance with the Helsinki Declaration. Informed consent was obtained from all of the patients participating in this study and was approved by the Local Ethics Committee.

### 4.1. CLL and Healthy Cell Isolation

For ex vivo experiments, a mixture of heparinized blood sample and Hanks’ Balanced Salt Solution (HBSS; Biomed, Lublin, Poland) 1:1 (*v*/*v*) was layered on the top of the Histopaque-1077 (Sigma Diagnostic, St. Louis, MO, USA) and centrifuged for 30 min at 200× *g*. The interphase region of peripheral blood mononuclear cells (PBMCs) was collected and washed twice in RPMI 1640, and then, cells were re-suspended in RPMI 1640 at a cell density of about 0.5 × 10^6^ cells per mL. A 1000-μL suspension of cells was placed into 24 culture well dishes (Nunc, Roskilde, Denmark).

### 4.2. Cell Cultures

Cell lines (Nalm-6, Raji, RPMI 8226, Toledo) and CLL leukemia cells were cultured in 10% RPMI 1640 medium containing heat-inactivated fetal calf serum (FCS) and antibiotics (streptomycin 50 mg/mL, penicillin 50 UI/mL: Life Technologies, Scotland). In the final experiments, cell cultures were incubated for 48 h, under standard conditions: 37 °C, 5% CO_2_, fully humidified. All of those experiments were repeated at least five times. 

### 4.3. Drug Dosing and Administration

Seven HAs were recently synthetized in the laboratory of Jack L. Arbiser and colleges, from Emory University School of Medicine, USA, and then studied in our laboratories. There following agents were used:

(1) BR MMK + phloroG-3,3′ dibromo4,4′ bis (dimethylamino) benzylidenephloroglucinol (HA 1),
(2) dibromoimipramine blue (HA 2),
(3) 3 bromo-4 dimethylamino diuhydroxyphenoxane (HA 3),
(4) bromo Gentian Violet-tribromogentian violet (HA 4),
(5) Gentian Violet (HA 5),
(6) BR dimethylaminobenzaldehyde + phloroG 3 bromo-4 dimethlyaminobenzylidenephloroglucinol (HA 6) and
(7) hexafluoro-diallylhexaflurobisphenol (HA 7).

### 4.4. Cytotoxicity and Apoptosis Assays

Overall cytotoxicity of HAs was estimated by using the propidium iodide (PI) flow cytometry assay. Namely, cells were washed twice with cold phosphate-buffered saline (PBS; Sigma Aldrich Chemie GmbH, Germany) after incubation and stained with PI at a concentration of 10 μg/mL, for 15 min, at room temperature (RT), in the dark. The half maximal inhibitory concentration (IC_50_) of the study drugs was estimated. 

Drug-induced apoptosis was determined by the annexin-V (Ann-V) assay. After incubation, cells were washed in cold PBS as mentioned above and then re-suspended in 100 μL of binding buffer containing 2 μL fluorescein isothiocyanate (FITC) conjugated Ann-V (Becton Dickinson, San Jose, CA, USA). Incubation was continued for 15 min, RT, in the dark. The fluorescence was measured by flow cytometry, using an FL1 standard fluorescent filter, at a 530 ± 20-nm wave-length. The compensated apoptotic index (CAI) was calculated as the difference in percent of Ann-V-positive cells in the drug-treated sample and parallel untreated culture. 

### 4.5. Drop of Mitochondrial Potential (*MTR, ΔΨ*)

For the assessment of the loss of mitochondrial membrane potential (ΔΨ), we used the MitoTracker Red 580 dye (Molecular Probes, Eugene, OR, USA). This assessment enabled us to distinguish the apoptotic from the non-apoptotic cell population. The stock solution of MitoTracker Red (1 mM) was diluted to achieve a working concentration of 50 nM and was added to the culture medium and incubated for 20 min at RT. ΔΨ was detected by flow cytometry (FL3 fluorescence filter).

### 4.6. Caspases and Apoptosis-Regulating Protein Expression

Activation of caspases-3, -8 and -9, as well as the expression of several apoptosis-regulating proteins, including the Bcl-2 family (Bax, Bak, Bcl-2, Mcl-1), the inhibitors of apoptosis protein (IAP) family (cIAP1, cIAP2, XIAP, Smac/DIABLO) and IAP antagonists (survivin, HTRA2/Omi), were also investigated.

FITC-conjugated monoclonal rabbit anti-active caspase-3 antibody was used to detect active caspase-3. Prior to the staining, cells were fixed and permeabilized by using Cytofix/Cytoperm TM solution. Permeabilization and fixation was conducted 20 min on ice, then washed and re-suspended in Perm/Wash TM buffer (all of those reagents were from BD Pharmingen, San Diego, CA, USA). After this time, the antibody was added in the concentration of 60 μL per 300 μL of cell suspension, and cells were stained for 30 min at RT. The green fluorescence of anti-active caspase-3 antibody was measured by flow cytometry using the FL1 filter after staining and washing in Perm/Wash TM buffer.

Caspase activation detected by fluorochrome-labeled inhibitors of caspase is now considered as a good marker of apoptosis [31]. Synthetic fluorochrome-labeled fluoromethyl ketone peptides (Immunochemistry Technologies LLC; Bloomington, MN, USA) bind to the active catalytic site of caspase proteases. The commercially available FAM-LETD-FMK FLICA™ Caspase-8 Assay Kit and FAM-LEGHD-FMA reagent-9 FLICA™ Caspase-9 Assay Kit were used for the assessment of active caspase-8 and caspase-9 following the protocol 150× concentrated solution in dimethylsulfoxide (DMSO; Sigma-Aldrich, St. Louis, MO, USA) that was initially prepared. 

Moreover, the expression of Bcl-2 family proteins (Bax, Bak, Bcl-2, Mcl-1), IAPs (cIAP1, cIAP2, XIAP, Smac/DIABLO) and IAP antagonists (survivin, HTRA2/Omi) was investigated by flow cytometry. Isolated PMBCs were fixed in 1% methanol free paraformaldehyde and permeabilized with 0.1% polysorbate 20 (Tween-20) in PBS (Amersham Bioscience, Freiburg, Germany). Cells were washed in PBS directly before incubation with the following Abs, commercially available: anti-human Bax primary rabbit antibody (Ab) in dilution 1:100, anti-Bcl-2 Ab in dilution 1:15 (all DAKO, Glostrup, Denmark), anti-Bak and anti-Bid primary rabbit anti-human monoclonal antibodies (MoAb) in dilution 1:10, mouse anti-Mcl-1 in dilution 1:30 (all from Abcam, Cambridge, UK) and MoAb anti-Akt (Becton Dickinson, San Jose, CA, USA) in dilution 1:15. For the assessment of the expression of IAPs and IAP antagonist protein, the following Abs were used: anti-cIAP1, anti-Smac/DIABLO, anti-survivin, rabbit polyclonal antibodies (PoAbs), as well as anti-cIAP2 and anti-XIAP goat PoAbs (all R&D Systems, Minneapolis, MN, USA) in a concentration of 1:100. All Abs were diluted in PBS containing 1% bovine serum albumin (BSA). Samples were incubated for 60 min, at RT, then washed in PBS by centrifugation (5 min 140× *g*). Next, secondary swine FITC-conjugated Ab anti-rabbit was used at a dilution of 1:20. Incubation was conducted for 60 min, at RT, in the dark. After this time, cells were washed and re-suspended in 400 μL PBS and analyzed. An increase or decrease of protein expression was compared to control and defined as up- or down-regulation, respectively. In regard to those protein expressions, the rates between MFI for treated and untreated samples were calculated.

### 4.7. Flow Cytometry Analysis

Viability, apoptosis and protein expression were analyzed by flow cytometry (FACScan: Becton-Dickinson, San Jose, CA, USA) using standard emission filters: FL-1: green for FITC (λ 530 ± 20 nm), FL-2: orange for R-PE (λ 564−606 nm), FL-3: red for Cy-5 (λ > 650 nm) and FL4: for APC (λ 800 ± 20 nm). The acquisition gate was established on FSC (forward scatter) and SSC (side scatter) that excluded dead cells and debris and included PMBCs. Ten thousand events were acquired for each analysis. For the assessment of apoptosis-regulating proteins, the level of mean fluorescence intensity (MFI) was assessed. 

### 4.8. Statistics

For the statistical analysis of the data, the range of the measured variables, means, medians and standard deviations (SD) were calculated, using statistical software (STATISTICA v.7.0, Tulsa, OK, USA). The data in the figures are presented as the median or mean ± SD values. The differences between values were evaluated with the non-parametric Mann–Whitney test. For the assessment of correlations, *p*-values less than 0.05 were considered statistically significant.

## Figures and Tables

**Table 1:** The half maximal inhibitory concentrations (IC_50_) induced in B-cell malignant cells by honokiol analogues (HAs).

Honokiol Analogues (HAs)	HA 1 IC_50_	HA 2 IC_50_	HA 4 IC_50_	HA 5 IC_50_
CLL	10 μM	5 μM	10 μM	5 μM
Raji	2.5 μM	2.5 μM	2.5 μM	0.25 μM
Toledo	0.5 μM	0.5 μM	2.5 μM	0.5 μM
RPMI 8226	0.5 μM	0.5 μM	5 μM	1 μM
Nalm-6	NR	NR	NR	NR

IC_50_, half maximal inhibitory concentration; NR, not reached; CLL, chronic lymphocytic leukemia cells; Raji, Burkitt lymphoma-driven; Toledo, diffuse large B-cell lymphoma; DLBCL, derived; RPMI 8226, multiple myeloma (MM)-driven; and Nalm-6, acute lymphoblastic leukemia (ALL)-derived cell lines.

**Table 2:** Expression of the examined proteins in particular cell types. The statistically-significant n-fold increases or decreases in mean fluorescence intensity (MFI) in HAs treated samples vs. untreated controls are shown.

**A. Apoptosis-Regulating Protein Expression in Response to HA1.**										
**Cells**	**HA1**									
	**Bax**	**Bak**	**Bcl-2**	**Mcl-1**	**c-IAP1**	**c-IAP2**	**XIAP**	**Smac-Diablo**	**Survivin**	**HTRA2/Omi**
CLL	NS	NS	0.78 *	0.51 **	0.59 **	0.47 **	0.27 ***	0.34 ***	1.68 *	NS
Raji	2.61 ***	2.12 **	0.39 ***	NS	NS	NS	NS	NS	1.73 *	NS
Toledo	2.93 ***	NS	0.49 **	NS	NS	NS	0.68 *	NS	2.06 **	NS
RPMI 8226	1.62 *	1.53 *	0.71 *	NS	NS	NS	0.75 *	NS	2.77 ***	NS
Nalm-6	NS	NS	0.82 *	NS	NS	NS	NS	NS	NS	NS
**B. Apoptosis-Regulating Protein Expression in Response to HA2.**										
**Cells**	**HA2**									
	**Bax**	**Bak**	**Bcl-2**	**Mcl-1**	**c-IAP1**	**c-IAP2**	**XIAP**	**Smac-Diablo**	**Survivin**	**HTRA2/Omi**
CLL	NS	NS	0.65 *	0.47 **	0.59 **	NS	0.29 ***	0.78 *	1.75 *	NS
Raji	2.33 ***	1.98 **	0.44 *	NS	NS	NS	NS	NS	1.67 *	1.92 *
Toledo	2.02 **	NS	0.35 ***	NS	NS	NS	0.52 **	NS	2.81 **	NS
RPMI 8226	1.50 *	NS	0.71 *	NS	NS	NS	0.73 *	NS	1.64 *	NS
Nalm-6	NS	NS	0.78 *	NS	NS	NS	NS	NS	NS	NS
**C. Apoptosis-Regulating Protein Expression in Response to HA4.**										
**Cells**	**HA4**									
	**Bax**	**Bak**	**Bcl-2**	**Mcl-1**	**c-IAP1**	**c-IAP2**	**XIAP**	**Smac-Diablo**	**Survivin**	**HTRA2/Omi**
CLL	NS	NS	0.72 *	0.38 *	NS	NS	0.29 ***	0.66 *	1.91 *	NS
Raji	2.29 *	1.94 *	0.59 *	NS	NS	NS	NS	NS	NS	1.87 *
Toledo	1.87 *	NS	0.42 *	NS	NS	NS	0.79 *	NS	2.85 ***	NS
RPMI 8226	1.76 *	NS	0.68 *	NS	NS	NS	0.73 *	NS	NS	NS
Nalm-6	NS	NS	NS	NS	NS	NS	NS	NS	NS	NS
**D. Apoptosis-Regulating Protein Expression in Response to HA5.**										
**Cells**	**HA5**									
	**Bax**	**Bak**	**Bcl-2**	**Mcl-1**	**c-IAP1**	**c-IAP2**	**XIAP**	**Smac-Diablo**	**Survivin**	**HTRA2/Omi**
CLL	NS	NS	0.55 **	0.65 *	0.72 *	NS	0.64 *	NS	0.42 **	NS
Raji	1.99 **	2.12 **	0.75 *	NS	NS	NS	NS	NS	0.68 *	NS
Toledo	2.23 **	NS	0.53 **	NS	NS	NS	0.44 **	NS	0.65 *	NS
RPMI 8226	1.68 *	1.80 *	0.72 *	NS	NS	NS	0.73 *	NS	0.73 *	NS
Nalm-6	NS	NS	NS	NS	NS	NS	0.60 *	NS	NS	NS

CLL, chronic lymphocytic leukemia; Raji, Burkitt lymphoma-derived; Toledo, diffuse large B-cell lymphoma; DLBCL, derived; RPMI 8226, multiple myeloma (MM)-derived; and Nalm-6, acute lymphoblastic leukemia (ALL)-derived cell lines; NS, not significant; *, **, *** statistically significant differences in protein expression in comparison to untreated control samples: * *p* < 0.05; ** *p* < 0.01; *** *p* < 0.001.
